# Reducing the need for colonoscopy with adjusted FIT and FOBT for stool weight and serum Hb levels

**DOI:** 10.3389/fmed.2023.1274024

**Published:** 2023-10-16

**Authors:** Niousha Ghomashi, Farzad Safari, Ali Noursina

**Affiliations:** School of Medicine, Isfahan University of Medical Sciences, Isfahan, Iran

**Keywords:** colonoscopy, hemoglobin, screening, colorectal cancer, fecal immunochemical test, fecal occult blood test, accuracy, sensitivity

## Introduction

Colorectal cancer (CRC) is a significant public health concern worldwide. Early detection and treatment of CRC can improve patient outcomes and reduce the disease burden. The fecal immunochemical test (FIT) and fecal occult blood test (FOBT) are non-invasive screening methods for CRC that can detect trace amounts of fecal hemoglobin (f-Hb), which may indicate the presence of CRC or advanced adenoma (1).

Many protocols to increase the accuracy of FIT/FOBT results have been investigated in studies. Adjusting for age and sex is one of the most straightforward approaches that can be used to increase the accuracy of these tests. Integration with other standard screening methods, such as flexible sigmoidoscopy, mtDNA testing, and CT colonography, is another approach to increasing the accuracy of FIT/FOBT results (2). In recent studies, integration with new methods, small RNA sequencing, and *Fusobacterium nucleatum* has been considered, methods that can detect false negatives and positives by checking the possibility of CRC from methods other than examining blood in the stool (3, 4). Although the protocols mentioned above are commendable, most studies have focused on increasing accuracy through integration with other methods. The knowledge gap in this field is that few studies have investigated improved methods for the FIT/FOBT tests.

## Research question

We hypothesized that adjusting for daily stool weight and serum Hb levels would improve the accuracy of FIT/FOBT results, which could lead to earlier detection of CRC. This protocol could reduce the added cost to the health system, as colonoscopy is a more costly method of CRC screening. The added cost of adjusting daily stool weight and serum Hb levels is negligible, and the potential benefits derived from the early detection and subsequent treatment of CRC far outweigh this expenditure.

## Hypothesizing method

We conducted a literature review to identify studies investigating the impact of daily stool weight and serum hemoglobin (Hb) levels on the accuracy of the fecal immunochemical test (FIT) and fecal occult blood test (FOBT) results.

Our literature review revealed no studies on the effect of daily stool weight on FIT or FOBT results. Most studies have focused on collection and post-collection techniques, which may influence f-Hb levels (5).

Research has shown that the accuracy of FIT and FOBT in stool samples can vary from day to day. This variation can be attributed to several factors, including differences in the intensity of bleeding from the neoplasm daily (6). We have put forth another possible explanation for the observed fluctuation: Daily stool weight can influence the precision of FIT and FOBT. We hypothesize that stool weight may influence f-Hb levels. The basis of our hypothesis is based on the phenomenon of dilution effects. Studies have demonstrated a correlation between the volume of intravascular fluid and the Hb levels in the blood. In the event of dehydration, the Hb levels in the blood may exhibit an elevated appearance, but this can be remedied through fluid therapy. This intervention produces a dilution effect, reducing the concentration of Hb in the blood (7). In a similar way, we hypothesize that individuals with higher daily stool weight are more likely to have a negative FIT or FOBT result, even if they have an advanced adenoma or CRC. A probable reason is that increased stool weight dilutes the concentration of f-Hb, making it more challenging to detect. Conversely, false-positive results may be observed in individuals with lower daily stool weight.

We found inadequate research on the impact of serum Hb levels on FIT/FOBT accuracy. While there have been studies on serum Hb levels and CRC, these mainly emphasize the importance of colonoscopy for patients with unexplained iron deficiency anemia (8, 9). D'Souza et al. (10) conducted a study that showed that patients with iron deficiency anemia and beta thalassemia trait may experience false-negative FIT results. However, the study did not explore the relationship between high serum Hb levels and the accuracy of FIT/FOBT (10). We hypothesize that serum Hb levels can affect FIT/FOBT accuracy because it can influence the amount of Hb that is present in the stool. If a person has low serum Hb levels, there may be less Hb in the stool, which could result in a false negative test result. Conversely, if a person has high serum Hb levels, there may be more Hb in the stool, which could result in a false positive test result.

## Proposed protocols

To improve the accuracy of FIT/FOBT results for colorectal cancer screening, we propose the following protocols based on efficiency, practicality, and lower cost:

1. Protocol one: adjusting FIT or FOBT results for sex, age, *Fusobacterium nucleatum*, and serum Hb levels.
2. Protocol two: adjusting FIT or FOBT results for sex, age, *Fusobacterium nucleatum*, serum Hb levels, and stool weight from which samples are taken;
3. Protocol three: adjusting FIT or FOBT results for sex, age, *Fusobacterium nucleatum*, serum Hb levels, and stool weight after bowel preparation and consuming a standardized food unit in which samples are taken.

These protocols offer different approaches to improving the accuracy of FIT/FOBT results. By adjusting test results for serum Hb levels, stool weight, and other factors, these protocols can help reduce false positives and negatives, leading to more accurate and reliable CRC screening. These protocols can be used according to the conditions to improve the accuracy of FIT/FOBT results.

These protocols are expected to have varying levels of patient adherence. Consequently, bowel preparation or stool weighing will not be mandatory for all individuals if the need for high accuracy is unnecessary and the individual is in the average-risk population. Protocols two and three are recommended if the patient needs a colonoscopy but has contraindications or if the patient does not need a colonoscopy but is in a high-risk population and has the necessary operation. Protocol one is recommended if the patient does not want to cooperate more or is in the average-risk population.

## Discussion

Our findings suggest that adjusting daily stool weight and serum Hb levels could improve the accuracy of FIT/FOBT results without significantly reducing their efficiency and practicality. This approach could lead to earlier detection and treatment of CRC, improving patient outcomes and decreasing the burden of CRC. If additional studies show that the proposed protocol can increase the accuracy of FIT/FOBT results to the point that it renders them a viable alternative to colonoscopy for screening, then it could be used for large-scale screening. This protocol would reduce the added cost to the health system without significantly increasing the risk of false-negative results.

To increase the efficiency and practicality of this protocol, adjusting FIT/FOBT results for serum Hb levels or stool weight could be done at tailored intervals in the high-risk or the general population. Although adjusting FIT or FOBT results for serum Hb levels and stool weight would reduce the efficiency and practicality of large-scale screening, it is crucial to consider that colonoscopy also has limitations in these areas.

Colonoscopy is a costly procedure and can be unpleasant. Patients must undergo bowel preparation, which can be time-consuming and distressing. In addition, there is a small risk of complications, such as bleeding or perforation. Some patients are reluctant to undergo colonoscopy because of cultural beliefs about the procedure or concerns about the risks (11, 12).

Even if the challenges before colonoscopy are overcome, there are still some challenges during the procedure. If bleeding or perforation occurs, they can terminate the procedure. In addition, if the bowel is not adequately prepared, the gastroenterologist may be unable to see lesions, leading to missed diagnoses (13).

In contrast, the proposed protocols for adjusting FIT/FOBT results for serum Hb levels and stool weight offer several potential benefits compared to colonoscopy. These protocols are non-invasive and do not require sedation. They are also less costly than colonoscopy and can be performed more frequently to improve early detection of CRC.

However, it is essential to note that these protocols also have limitations. Adjusting FIT/FOBT results for serum Hb levels and stool weight may not detect all cases of CRC or advanced adenoma. The proposed protocol may be a more efficient and practical screening method than a colonoscopy. However, further studies are needed to confirm its accuracy and compare its benefits and limitations to other screening methods.

## Conclusion

Factors such as daily stool weight and serum Hb levels can affect the accuracy of FIT/FOBT results. These factors can lead to false-negative and false-positive results, which can delay the diagnosis and treatment of CRC. To improve the accuracy of FIT/FOBT results, patients could adhere to standard procedures before taking the test. In addition, investigating new protocols for adjusting daily stool weight and serum Hb levels to improve the accuracy of these tests is needed. Early detection and treatment of CRC is essential for improving patient outcomes. We can decrease the burden of CRC by improving the accuracy of FIT/FOBT results without significantly reducing their efficiency and practicality.

## Limitations and future research

It is important to note that this study presents a theoretical protocol and hypothesis, and further research is needed to confirm our findings. Future studies could investigate the impact of daily stool weight and serum Hb levels on FIT/FOBT results accuracy using various study designs such as cross-sectional retrospective prospective and randomized clinical trials.

## Author contributions

NG: Writing—original draft, Writing—review and editing. FS: Conceptualization, Investigation, Methodology, Project administration, Resources, Software, Supervision, Validation, Visualization, Writing—original draft, Writing—review and editing. AN: Conceptualization, Investigation, Methodology, Project administration, Resources, Software, Supervision, Validation, Visualization, Writing—original draft, Writing—review and editing.
